# Investigating Concordance among Genetic Data, Subspecies Circumscriptions and Hostplant Use in the Nymphalid Butterfly *Polygonia faunus*

**DOI:** 10.1371/journal.pone.0041058

**Published:** 2012-07-23

**Authors:** Ullasa Kodandaramaiah, Elisabet Weingartner, Niklas Janz, Michael Leski, Jessica Slove, Andrew Warren, Sören Nylin

**Affiliations:** 1 Department of Zoology, Stockholm University, Stockholm, Sweden; 2 Independent Consultant, Buffalo Grove, Illinois, United States of America; 3 McGuire Center for Lepidoptera and Biodiversity, Florida Museum of Natural History, University of Florida, Gainesville, Florida, United States of America; University of Oxford, United Kingdom

## Abstract

Subspecies are commonly used taxonomic units to formally describe intraspecific geographic variation in morphological traits. However, the concept of subspecies is not clearly defined, and there is little agreement about what they represent in terms of evolutionary units, and whether they can be used as reliably useful units in conservation, evolutionary theory and taxonomy. We here investigate whether the morphologically well-characterized subspecies in the North American butterfly *Polygonia faunus* are supported by genetic data from mitochondrial sequences and eight microsatellite loci. We also investigate the phylogeographic structure of *P. faunus* and test whether similarities in host-plant use among populations are related to genetic similarity. Neither the nuclear nor the mitochondrial data corroborated subspecies groupings. We found three well defined genetic clusters corresponding to California, Arizona and (New Mexico+Colorado). There was little structuring among the remaining populations, probably due to gene flow across populations. We found no support for the hypothesis that similarities in host use are related to genetic proximity. The results indicate that the species underwent a recent rapid expansion, probably from two glacial refugia in western North America. The mitochondrial haplotype network indicates at least two independent expansion phases into eastern North America. Our results clearly demonstrate that subspecies in *P. faunus* do not conform to the structuring of genetic variation. More studies on insects and other invertebrates are needed to better understand the scope of this phenomenon. The results of this study will be crucial in designing further experiments to understand the evolution of hostplant utilization in this species.

## Introduction

The concept of species, linked intrinsically to the process of speciation, has been the source of long-standing debate of an intensity matched by few others in biology. Lineages within a species that are presumably under various stages of speciation have been an integral part of this discussion. From a taxonomic perspective, the intraspecific category representing diverging lineages within a species is often the subspecies [1]. Patten and Unitt [2] define subspecies as “a collection of populations occupying a distinct breeding range and diagnosably distinct from other such populations”, a definition generally accepted in practice. Subspecies have traditionally been circumscribed based on discontinuities in the geographical distribution of phenotypic traits [3], which has sometimes resulted in highly subjective delimitations. It is common practice in taxonomic revisions for several closely related taxa to be lumped together as subspecies, or subspecies to be elevated to the level of species. It follows that various taxa currently considered subspecies might represent *bona fide* species. Indeed, it has been suggested that trinomial names in collections should be maintained since some subspecies may represent true species [4]. Apart from their relevance in taxonomy, they are commonly used as tools in biodiversity assessment and to study evolutionary divergence even though their relevance in biology has been extensively debated. On the one hand they have been postulated to represent ‘incipient species’ [5] that could eventually evolve into distinct species over time, while on the other hand some authors have argued that they are the only taxonomic unit inconsistent with evolutionary history [6]. In summary, despite the arbitrary taxonomic nature of subspecies, they have been vital units in classification, conservation planning and evolutionary theory [7].

Some authors consider that the majority of *bona fide* subspecies are monophyletic [8], and monophyly has frequently been used to test their validity [7], [9]. A large-scale meta analysis [7] reported that 97% of avian subspecies were not supported by mitochondrial DNA monophyly, and concluded that subspecies mislead conservation policy and evolutionary studies. In contrast, a more comprehensive study [9] reported that at least a third of the subspecies in birds were phylogenetically distinct. They concluded that subspecies can often serve as proxies for the estimation of intraspecific genetic diversity, and therefore lend themselves as a useful tool in studying evolutionary divergence and conservation planning. Nevertheless, testing the validity of subspecies using monophyly as a criterion based on genetic data is questionable since subspecies are not necessarily reproductively isolated. By definition, two subspecies distributed in parapatry have some degree of gene flow between them. Furthermore, there may not have been enough time for divergence and reciprocal monophyly to evolve. Patten [10] hence advocates using less restrictive clustering-based methods to ascertain their credibility. Studies using such an approach have been very few, especially in the case of invertebrates (e.g. [11]). Given the practical importance of subspecies in various fields of biology, it is imperative that subspecific circumscriptions are vigorously examined in more polytypic species, particularly within invertebrates.

Mitochondrial DNA is widely used to examine species relationships, and less often for subspecific relationships. The use of mitochondrial DNA in invertebrate taxonomy is complicated by potential indirect selection on the mitochondrial genome by cytoplasmic endosymbionts such as *Wolbachia* [12]. *Wolbachia* alone is estimated to have infected up to three-fourths of all insect species [13]. These microorganisms (mostly bacteria) are maternally inherited, and have evolved a suite of mechanisms - male-killing, cytoplasmic incompatibility and feminization - which confer a selective advantage to individuals infected by the symbionts [14]. Thereby, they have the potential to spread rapidly in the host population, with mitochondrial haplotypes hitch-hiking along and eventually leading to drastic loss of genetic diversity. Indeed, this is the case in several insect species (see [12] for a review). Furthermore, introgression between subspecies, when accompanied by *Wolbachia*, can quickly replace mitochondrial haplotypes in uninfected subspecies. Therefore, relying solely on mitochondrial data to study subspecies in invertebrates is not justified.

We here use a holistic approach based on data from three sources - mitochondrial sequences, 8 microsatellite loci and molecular assays to detect *Wolbachia* infection - to investigate whether described subspecies in the well-known butterfly species *Polygonia faunus* (Green Anglewing) are genetically distinct. Given the problems with using mitochondrial monophyly as the sole criterion to test the validity of subspecies [10], we employ a Bayesian clustering-based method in addition to standard monophyly and distance-based methods. This species is especially interesting for such an exercise because most subspecies are well-characterized and ecologically distinct. Ecological traits differing among subspecies in this polyphagous butterfly include hostplant utilization. Comparative work has indicated that polyphagy in herbivorous insects promotes diversification [15], [16]. Experimental work on its sister species - *P. c-album* - has also shown that differences in hostplant preferences are at least partly genetically determined [17], [18]. Therefore, in the light of the differences in hostplant preference across subspecies, the question arises whether *P*. *faunus* subspecies represent incipient species in intermediate stages of ecological speciation. To answer this, it is paramount to first determine whether these subspecies have a genetic underpinning or if the morphological differences are largely geographic variations expressed due to phenotypic plasticity. The molecular data are also crucial in understanding the population structure and phylogeographic history of the species, thereby gaining a deeper understanding of the evolution of subspecies (or genetic populations) and hostplant preferences. This information also forms the basis for an interesting comparison with the sister species for which similar data have become recently available [19].

### The Study Species

**Figure 1:**
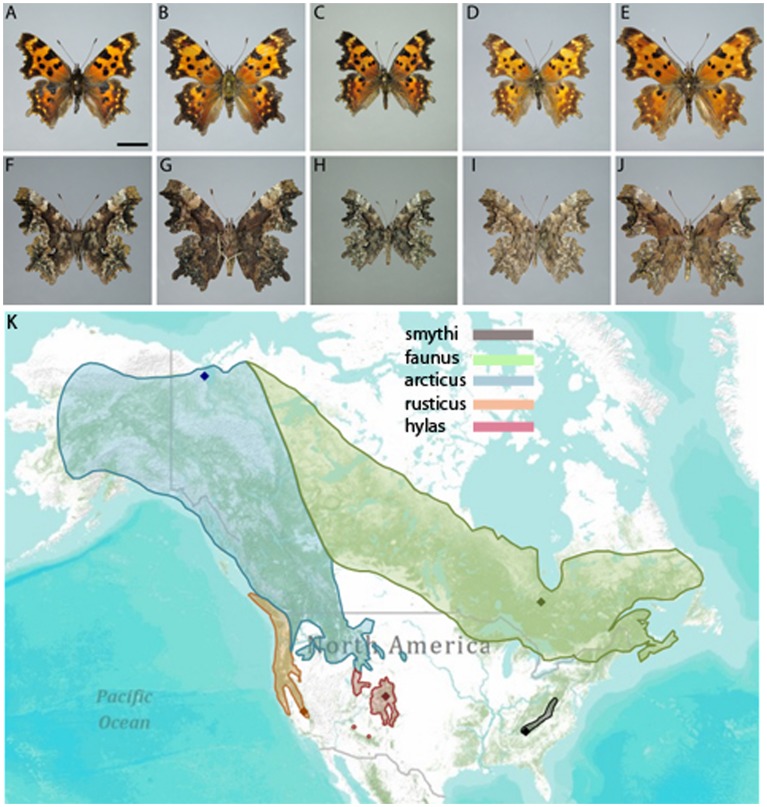
Subspecies of *Polygonia faunus* and distribution map. Shown are representative photographs of the Polygonia faunus subspecies: a) ‘faunus’, 8 miles northwest of Fraserdale, Ontario; b) ‘smythi’, Cooper Creek Wildlife Management Area, 11 miles southeast of Blairsville, Georgia; c) ‘arcticus’, Bug Creek, 15 miles southwest of Aklavik, NWT, Canada; d) ‘hylas’, 0.25 miles west of Turquoise Lake Reservoir, Leadville, Colorado; and e), ‘rusticus’, Lewis Creek, Sugar Pines, California. Fresh vouchers of subspecies ‘fulvescens’ were unavailable for this study. The distribution map of each subspecies is illustrated in f). Diamonds indicate the location of the specimens shown in a-e). A blend zone exists at the ‘rusticus’/’arcticus’ interface, and probably at the ‘faunus’/’arcticus’ and ‘arcticus’/’hylas’ interfaces (not illustrated).

*Polygonia faunus* occurs in woodlands, damp mountain meadows and stream sides, and is often abundant in boreal habitats. The larvae feed on plants from the families Betulaceae, Ericaceae, Grossulariaceae and Salicaceae in the wild, but have also been shown to be able to feed on Urticaceae in the laboratory [20]. Six subspecies are most commonly recognized [21] (Figure 1): ‘faunus’ distributed in northeastern United States and across eastern Canada into western Canada where it probably blends into ‘articus’; ‘smythi’ in the southern Appalachian Mountains; ‘arcticus’ from a blend zone in northwestern United States and southwestern Canada where it blends into ‘rusticus’, western Canada and Alaska; ‘hylas’ in the southern Rocky Mountain region; ‘rusticus’ in California to Vancouver Island, Canada; and ‘fulvescens’ in coastal northern California. Known hostplant use of the different subspecies are as follows: *arcticus* feeds on *Salix; hylas* on *Salix* and *Ribes; smythi* on *Betula,*and *rusticus* on *Rhododendron,* although larvae may accept others in the lab [22], [23].

### Objectives of the Study

1. To investigate the corroboration between genetic groupings and subspecies in *P. faunus*.
2. Test whether patterns of hostplant use are correlated with genetic clustering.
3. Compare the phylogeographic structure and potential effect of *Wolbachia* in *P*. *faunus* to that of its sister species *P*. *c-album* where *Wolbachia* is highly prevalent and appears to have had an impact on the diversity of its mitochondrial DNA.

## Results

### Mitochondrial DNA

There were 38 polymorphic sites and 35 unique haplotypes in the 1422 bp COI (cytochrome oxidase subunit I) dataset. Global haplotype diversity was 77.29% (SD ±2.7%); values for each population are given in Table 1. Gobal Ф_ST_ value was 38.16 (P = 0.00) and pairwise Ф_ST_ values of population pairs are shown in Table 2. Most populations were significantly differentiated from others (bold values in Table 2). All subspecies pairs were significantly differentiated (Table 3).

**Table 1:** Number of mitochondrial haplotypes and haplotype diversity values of each population.

Population	No. of haplotypes (samples)	Haplotype diversity (H)
British Columbia (BCo)	3 (19)	0.2047+/−0.1191
Washington (Was)	4(12)	0.4545+/−0.1701
Alberta (Alb)	5(7)	0.8571+/−0.1371
Montana (Mon)	5(16)	0.4500+/−0.1507
Colorado (Col)	8(28)	0.5397+/−0.1105
Utah (Uta)	7(22)	0.8636+/−0.0786
Arizona (Ari)	1(9)	0
New Mexico (NMe)	1(6)	0
New Hampshire (NHa)	4(5)	0.9000+/−0.1610
California (Cal)	4(8)	0.7500+/−0.1391
Oregon (Ore)	2(6)	0.5333+/−0.1721

Idaho, Quebec and Georgia were excluded from the analysis since they were represented by less than five individuals.

**Table 2:** Pairwise Ф_ST_ values between populations calculated from mitochondrial haplotype frequencies in Arlequin.

	BCo	Was	Alb	Mon	Col	Uta	Ari	NMe	NHa	Cal
Was	−0.02									
Alb	**0.80**	0.74								
Mon	0.63	0.60	−0.06							
Col	**0.71**	**0.62**	**0.48**	**0.46**						
Uta	**0.14**	**0.03**	0.51	**0.48**	**0.43**					
Ari	**0.75**	**0.68**	**0.75**	0.76	0.66	**0.58**				
NMe	**0.26**	**0.20**	**0.09**	**0.19**	0.27	0.10	0.53			
NHa	**0.18**	**0.19**	0.68	**0.59**	**0.51**	0.13	**0.60**	**0.19**		
Cal	**0.82**	**0.77**	**0.00**	−**0.03**	**0.57**	**0.58**	**0.80**	**0.15**	**0.74**	
Ore	0.15	0.00	0.74	0.57	**0.47**	−0.10	**0.57**	**0.10**	0.11	**0.80**

**Figure 2:**
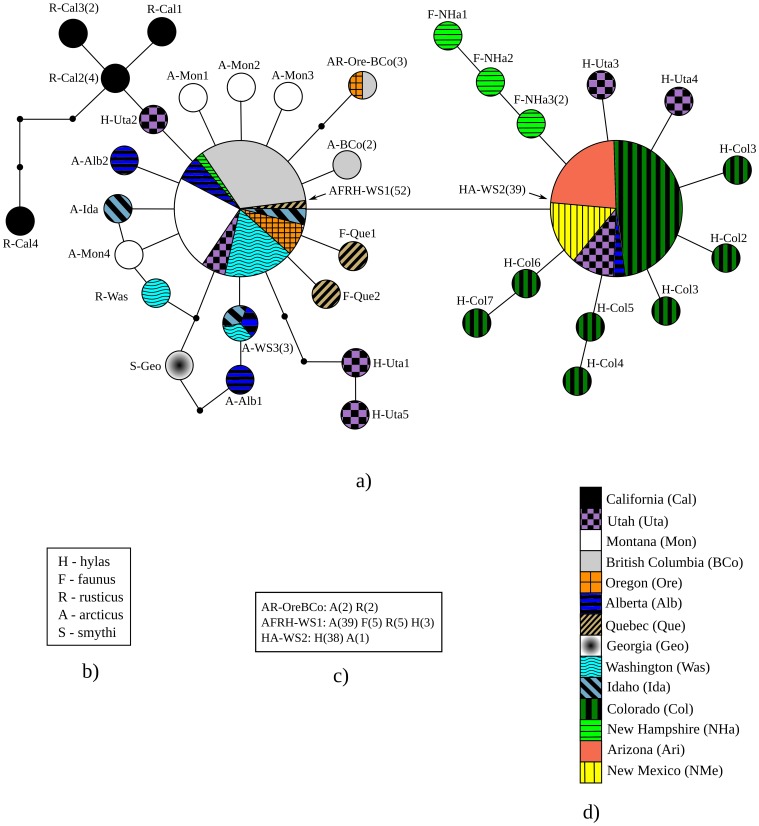
The statistical parsimony network of 35 *Polygonia faunus* mitochondrial haplotypes identified in the study, reconstructed using the software TCS v1.21. Each circle represents a haplotype and is approximately proportional in area to the number of individuals possessing the haplotype. The smallest circles represent missing haplotypes. Each haplotype is named using the following convention: The alphabets preceding the hyphen indicate the subspecies as listed in b), and the alphabets following the hyphen indicate the populations in which the haplotype was recovered, with each population abbreviated according to the list in d). Widespread haplotypes, i.e., those occurring in more than two populations, have a ‘WS’ after the hyphen. For the three haplotypes found in more than one subspecies, c) lists the numbers of individuals for each; d) is the legend to the patterns representing each population on the network.

Values significantly different in the exact tests of differentiation are in bold. Population abbreviations are as in Table 1 and Figure 2.

**Table 3:** Pairwise Ф_ST_ values between subspecies calculated from mitochondrial haplotype frequencies in Arlequin.

	arcticus	hylas	faunus
hylas	**0.42**	0.00	
faunus	**0.42**	**0.31**	0.00
rusticus	**0.52**	**0.29**	**0.55**

Values significantly different in the exact tests of differentiation are in bold.

**Figure 3:**
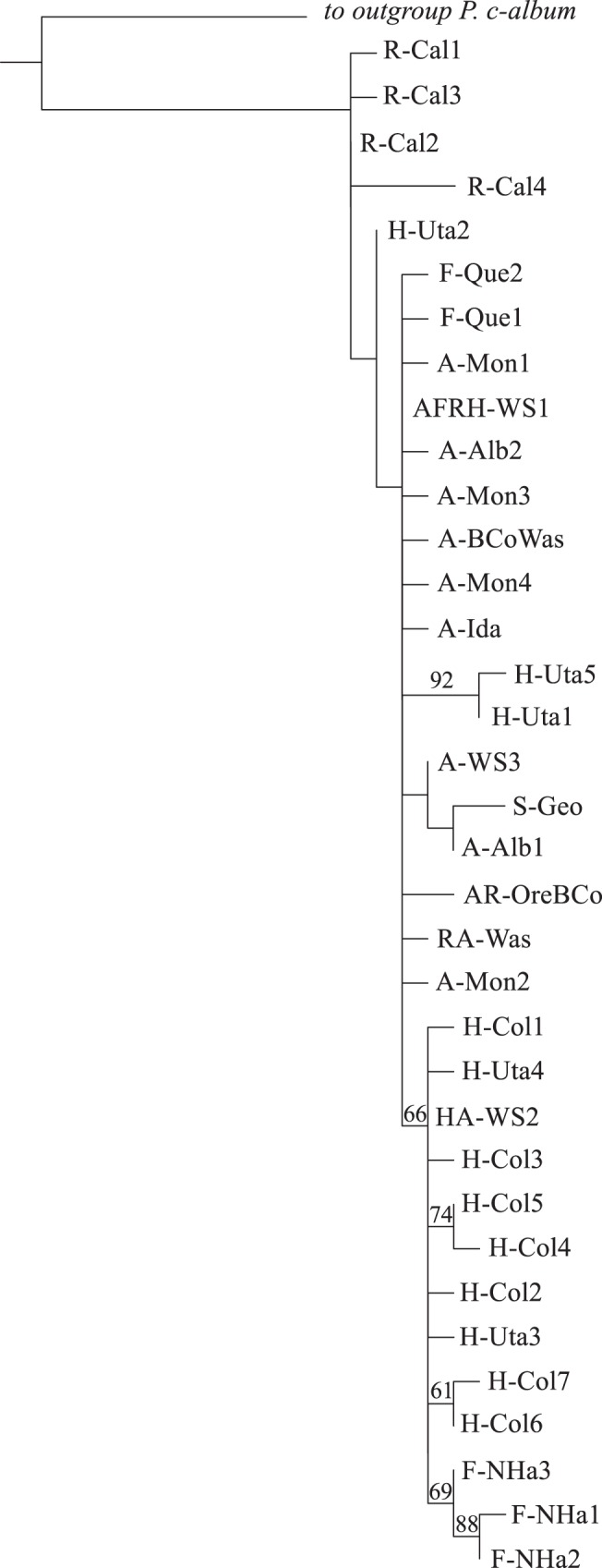
Maximum Likelihood phylogeny of mtDNA haplotypes inferred in RaXML. Numbers above branches are bootstrap support values greater than 50%. The length of branch leading to the outgroup is not to scale.

The statistical parsimony network of haplotypes is depicted in Figure 2, with each haplotype labeled to facilitate interpretation and discussion. Two central haplotypes AFRH-WS1 and HA-WS2 were the most widespread, occurring in nine and four populations respectively. Twenty-six haplotypes were restricted to single individuals. The phylogeny of haplotypes was largely unresolved (Figure 3).

The mismatch distribution curve had a negative slope and did not deviate from that expected under a model of sudden expansion (Sum of Squared deviation = 0.00210305; P (Simulated sum of Squared Deviation > = Observed Sum of Squared Deviation) = 0.21800000). In the Fu’s Fs test of selective neutrality, the simulated Fs value was significantly lesser than the observed value (Fs = −28.08451; Prob (simFs< = obsFs) = 0.00000).

### Microsatellites

The number of alleles per locus ranged from four to 22 (Table 4). Observed heterozygosity values for four loci were significantly lower than expected. Populations represented by fewer than five individuals were excluded from pairwise comparisons. Pairwise F_ST_ values are presented in Table 5, with significant values in bold; most populations were significantly differentiated from each other. Table 5 also includes pairwise F_ST_ values estimated after correcting for the presence of null alleles. Table 6 depicts pairwise F_ST_ between subspecies pairs, both with and without ENA correction in the software FreeNA.

**Table 4:** Allelic variability, expected and observed heterozygosity values estimated from genotyping 137 *Polygonia faunus* individuals for 8 microsatellite loci.

Locus	Repeat	Allelic range	No. of alleles	Obs. Het	Exp.Het
*Prim2*	2	136–146	6	0.47368	0.63727
*Prim5*	4	201–297	9	**0.05263**	0.74964
*Prim6*	2	91–137	8	**0.52632**	0.81081
*Prim7*	2	164–218	10	0.68421	0.79516
*Prim8*	2	116–140	10	0.89474	0.83926
*Prim11*	4	193–217	2	0.21053	0.27312
*Prim17*	2	87–97	5	**0.52632**	0.73826
*Prim20*	2	174–230	8	**0.36842**	0.78947

Observed heterozygosity values significantly lower than expected are in bold. The second column lists the lengths of repeat units for each locus.

**Table 5:** Pairwise F_ST_ values between populations calculated from the microsatellite data.

	BCo	Was	Alb	Mon	Col	Uta	Ari	NMe	NHa	Cal	Ore
BCo	N/A	0.01	−0.01	0.00	0.06	0.03	0.17	0.14	0.01	0.13	−0.01
Was	0.01	N/A	0.00	0.00	0.08	0.02	0.20	0.15	0.03	0.14	−0.01
Alb	0.00	0.02	N/A	−0.01	0.06	0.04	0.21	0.16	0.03	0.14	−0.01
Mon	0.01	0.01	0.02	N/A	0.07	0.03	0.20	0.15	0.00	0.15	0.00
Col	**0.07**	**0.09**	**0.08**	**0.08**	N/A	0.09	0.12	0.07	0.09	0.19	0.07
Uta	**0.04**	0.03	**0.07**	**0.05**	**0.10**	N/A	0.22	0.16	0.03	0.13	0.03
Ari	**0.18**	**0.22**	**0.24**	**0.23**	**0.14**	**0.24**	N/A	0.17	0.26	0.28	0.19
NMe	**0.15**	**0.16**	**0.18**	**0.16**	**0.08**	**0.18**	**0.21**	N/A	0.15	0.31	0.13
NHa	0.02	0.04	0.05	0.02	**0.10**	**0.06**	**0.30**	**0.18**	N/A	0.18	0.01
Cal	**0.13**	**0.14**	**0.15**	**0.18**	**0.19**	**0.13**	**0.28**	**0.32**	**0.20**	N/A	0.15
Ore	−0.01	0.00	0.00	0.01	**0.07**	**0.06**	**0.22**	**0.16**	0.04	**0.17**	N/A

Numbers to the left of the diagonal are calculated in Arlequin without correcting for the presence of null alleles. Values in bold are significantly greater than zero. Numbers to the right of the diagonal are F_ST_ values calculated in FreeNA after ENA correction for null alleles. Population abbreviations are as in Table 1 and Figure 2.

**Table 6:** Pairwise F_ST_ values between subspecies calculated from the microsatellite data.

	arcticus	hylas	faunus	rusticus
arcticus	N/A	0.05	0.01	0.04
hylas	**0.06**	N/A	0.08	0.07
faunus	0.01	**0.08**	N/A	0.07
rusticus	**0.04**	**0.08**	**0.09**	N/A

Numbers to the left of the diagonal are calculated in Arlequin without correcting for the presence of null alleles. Values in bold are significantly greater than zero. Numbers to the right of the diagonal are F_ST_ values calculated in FreeNA after ENA correction for null alleles.

**Figure 4:**
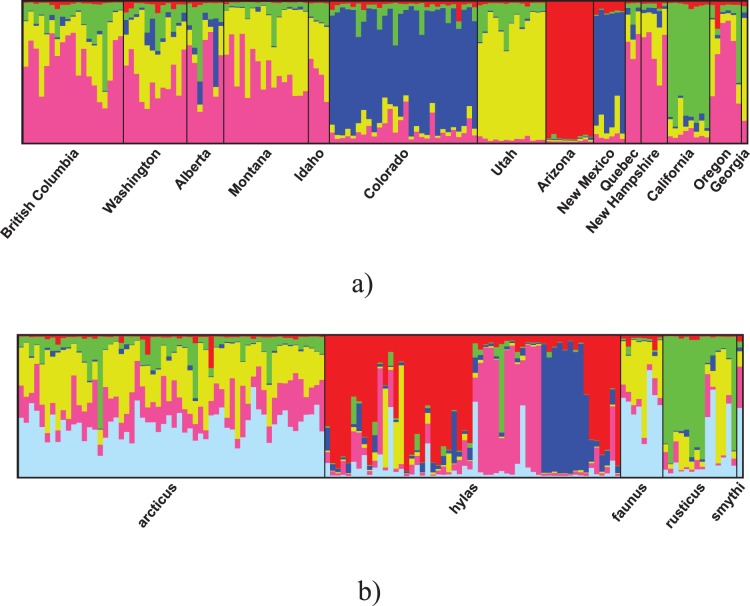
Population structure inferred in STRUCTURE based on microsatellite data. Each genetic cluster is represented by a colour. Every individual is represented by a single vertical line with coloured segments depicting the estimated proportion of ancestry from a given cluster. a) Results from the analysis where individuals were grouped into populations *a priori*; K = 5. b) Results from the analysis where individuals were grouped into subspecies *a priori*; K = 6.

In the first STRUCTURE analysis where individuals were grouped according to populations *a priori*, the log likelihood peaked at K = 5. The series of analyses with K = 1 to 14 was repeated five times to determine whether the likelihood was consistently highest at K = 5. The best value of K ranged between five and seven. However, all analyses with K = 4 to 7 recovered four well-defined clusters - Arizona, California, Utah and New Mexico+Colorado (Figure 4a). These clusters were progressively less cohesive as K increased beyond 7. In the analysis where individuals were grouped into subspecies *a priori*, the highest likelihood was at K = 6. None of the clusters corresponded to a subspecies (Figure 4b). Results with the dominant markers model imposed gave similar results.

### *Wolbachia* Assays

11 samples (8%) tested positive for *Wolbachia* infection. These individuals were from California (2), Colorado (2), Utah (3), Arizona (1), Washington (2) and Montana (1).

## Discussion

### Subspecies in *P. faunus*

Although results from the mitochondrial and microsatellite data were not completely congruent, neither dataset corroborated subspecific groupings in *P. faunus*. Ф_ST_ and F_ST_ values indicate that most populations and subspecies are differentiated from other populations and subspecies respectively. The haplotype network, phylogeny and the clustering analysis in STRUCTURE all indicate strong structuring of genetic variation in the species, but none of these genetic groupings corresponds to subspecies. Furthermore, we found little evidence for geographic structuring based on hostplant use patterns. We conclude that the morphological and ecological differences among subspecies in *P. faunus* are either phenotypically plastic traits expressed differentially across populations or local adaptations with genetic underpinnings that evolve rapidly. The use of different hostplants may also be related to differences in availability *per se*. We note that current knowledge of host use across the range of the species is based mainly on isolated findings, and hence the information available is at a coarse scale. The species is ideally suited for detailed experimental work to understand the factors determining the evolution of female and larval host choice. The results from the current study will prove invaluable in designing such experiments.

### How Useful are Subspecies in Biology?

This study is one of the first to explicitly test the validity of subspecies in an insect species using a combination of markers and analysis methods. Other studies have similarly reported a lack of support for subspecific groupings (e.g. [11], [24]). Ecological and morphological variation across populations is better characterized in *P. faunus* compared to many other insect species, and typical insect subspecies are circumscribed on the basis of less distinct features (although morphological and ecological distinction between ‘rusticus’ and ‘arcticus’ is not very clear because of a broad blend zone that extends from the Cascade Mountains in Washington into Idaho, Montana, and southern British Colombia and Alberta). Taxonomists have frequently used highly variable and/or plastic characters to describe subspecies, an excellent example being the use of eyespot (ocelli) number in satyrine butterflies. The number of eyespots has been shown both to evolve rapidly under selection, and to be highly plastic (see [11]). However, in the absence of detailed experimental work, it is often impossible to distinguish plastic traits from those that have evolved over time in specific localities. Common garden experiments on *P*. *faunus* subspecies to test the extent of plasticity in wing patterns will be illuminating.

Our results point to an interesting conflict with regard to the evolutionary significance of subspecies in *P. faunus*. On the one hand, our results suggest that these and most currently described subspecies in insects and other invertebrates need to be critically re-evaluated, preferably using genetic data. There may be several cases where subspecies represent distinct evolutionary lineages, perhaps even cryptic species. However, unless corroborated by genetic data, these “morphotypes” can only be regarded as phenotypic forms over spatial scales, and not as evolutionary units of importance. Even though generating genetic data to determine subspecific validity for all species of interest is highly impractical, results thus far suggest that it is dangerous to use subspecies as proxies for evolutionary units in biodiversity assessment, ecology and evolution.

On the other hand, it is still quite interesting to note that there can be such phenotypically distinct “morphotypes” in different parts of a geographic range that do not have an obvious genetic foundation. This means that these populations express different parts of a common reaction norm, which is a prerequisite for divergent selection. One example of this differential distribution is displayed by the distinct ‘smythi’ morphotype, which occurs sporadically throughout the range of *faunus*, from northern NH to eastern Saskatchewan (MLL, unpublished observations). Another example is given by the ‘faunus’ and ‘arcticus’ morphotypes, which are sympatric at several of the locations sampled for this study (for example, 70 km south of Inuvik on the Dempster Highway in Northwest Territories) and probably across a broad range of western Canada (see Figure 1), although this region is difficult to sample and rarely frequented by lepidopterists when *P. faunus* is in flight. Despite these examples, we note that the salient features of the individual subspecies are apparent in large series. We speculate that the expression of these morphotypes is influenced by the local ground coloration, as *P. faunus* adults feed primarily on moist soil and animal scat. Thus, the ventral coloration of ‘faunus’ and ‘smythi’ morphotypes may aid crypsis on the darker eastern soils, that of ‘arcticus’ readily blends into the mossy ground colors of northwestern Canada, and ‘hylas’ and ‘rusticus’, as the latter name implies, nicely mimic the iron-rich surfaces of the western United States. Selection can only act on existing phenotypic variation and these morphotypes may in fact be quite important for setting the stage and providing opportunities for divergent selection and possible future genetic divergence (c.f. [25], [26]). Although subspecies that lack a genetic foundation are highly questionable as evolutionary units in themselves, they may thus still hold evolutionary importance, as raw material for future evolution.

### Population Structure

The Californian population represents the southernmost range of the species west of the Rockies. This population is isolated by the Great Basin separating the Sierra Nevada Mountains from the Rocky Mountains. Not surprisingly, it was distinct in both the haplotype network and the STRUCTURE analysis.

Interestingly, Arizona, the southernmost population, was strongly differentiated in the STRUCTURE analysis, but all individuals from this population had a single haplotype shared with Colorado, Utah and New Mexico. Given the low prevalence of *Wolbachia*, we rule out the effect of selective sweeps related to the bacterium as an explanation for this discordance between mitochondrial and nuclear data. Microsatellites are generally more variable than mitochondrial sequences and hence more likely to reflect recent changes in population structure. We therefore suggest that Arizona has been recently isolated from other populations and has since evolved in isolation. Our personal observations indicate that individuals in Arizona are on average larger and more richly colored on the ventral side compared to other *hylas* from the Rockies.

Colorado and New Mexico together were recovered as a well-defined group by STRUCTURE, whereas this group was not cohesive in the haplotype network. Other populations were heterogeneous in origin in both the network and STRUCTURE results. This could be due to higher levels of gene flow among these populations, or simply because these regions were colonized very recently.

The low-lying, arid Wyoming Basin is thought to be a barrier for dispersal in high-elevation taxa [27]–[31]. The two haplotype groups, separated by the widespread haplotypes AFRH-WS1 and HA-WS2, may reflect structuring due to a genetic barrier imposed by the Wyoming Basin.

### Phylogeography

Our data suggest that the species has undergone a recent and rapid population expansion, probably following the last glacial maximum. If this indeed was the case, the two star-shaped haplotype groups likely reflect expansion from two refugia. Utah, which includes haplotypes closely related to populations both east and west of the Rocky mountain cordillera, could be part of the region of contact between the two waves of expansion.

The New Hampshire population has haplotypes from both major haplotype groups, indicating at least two waves of colonization into the east. The species is rare in the southeastern USA and we were unfortunately only able to sample a single individual from Georgia, which represents the south-eastern extreme of distribution. The extant distribution indicates that it is likely that the species reached Georgia from the north through the Appalachian Mountains, and not directly from the Rocky Mountains. Further studies with a more comprehensive sampling of populations in the eastern part of the continent will be interesting.

### Comparison with *P. c-album*

*P. faunus* exhibits a higher haplotype (77.29% versus 40.25%) diversity in mitochondrial DNA compared to *P. c-album*. The mitochondrial population structure found in *P. faunus* was deeper compared to that in its sister species. Results in [19] strongly indicate that a selective sweep related to *Wolbachia* infection has drastically reduced the diversity and obliterated population structure of the mitochondrial genome in *P. c-album*. However, the prevalence of *Wolbachia* in *P. faunus* is restricted to a small percentage of individuals. It is therefore unlikely that *Wolbachia* has affected mitochondrial structure in *P. faunus* to a strong degree.

Since the microsatellite markers were developed for *P. c-album*, gene diversity (i.e. expected heterozygosity) cannot be compared across the two species. The STRUCTURE analysis in *P. c-album* indicated that two populations - Morocco and Russia - were genetically distinct, while there was no structure within the European populations. The findings in *P. faunus* are similar in that there were three distinct genetic clusters, but very limited structure among the remaining populations. Although hostplant use is known to vary across populations in both species, neither study supports the hypothesis that these differences are related to gene flow between populations. Therefore, until there is experimental evidence that supports a genetic basis for the differences in host use, differences among populations should be seen as potentially being plastic responses to varying environmental conditions, chief among which is availability of a particular host species.

### Summary and Conclusions

We have used data from a combination of mitochondrial sequences and eight microsatellite loci to study the population genetics and phylogeography of *P. faunus*. We tested whether subspecies definitions are supported by the genetic data, but find no evidence for this. We instead found that there are three distinct genetic clusters in the species - California, Arizona and New Mexico+Colorado. There was little structuring among the remaining populations, probably due to gene flow across populations. Our results suggest that subspecies in insects need critical re-evaluation, preferably with genetic data, before being considered as important units in biology. However, it is interesting that such phenotypically distinct and geographically separated morphotypes are not supported by genetic data. Further work is needed to understand the evolution of such phenotypes. We found no support for the idea that broad differences in host use are correlated with genetic distance between populations. The results indicate that the species underwent a recent rapid expansion, probably from two glacial refugia in western North America. The haplotype network also indicates at least two independent expansion phases into eastern North America. Results in this study will be crucial in designing further experiments to understand the evolution of hostplant utilization in this species.

## Materials and Methods

### Sampling and DNA Extraction

137 samples representing 14 populations - British Columbia, Washington, New Mexico, Colorado, Quebec, New Hampshire, Alberta, California, Utah, Montana, Oregon, Arizona, Idaho and Georgia – were collected by us mainly between 2000 and 2007 (Figure 1; Additional File 1). All necessary permits were obtained for the described field studies. These were only required for the Yukon and Northwest Territory. Research in the Yukon was supported by License No.11-10S&A (Jeff Hunston, Manager, Heritage Resources Unit, Cultural Services Branch, Department of Tourism & Culture, Government of Yukon, Box 2703, Whitehorse, Yukon Y1A 2C6). Research in the Northwest Territories was supported by Scientific Research License # 14902 (Jonathon Michel, Manager, Scientific Services, Aurora Research Institute, PO Box 1450, 191 Mackenzie Road, Inuvik, NT, X0E 0T0) and also by a Research Agreement for Vuntut Gwitchin Settlement Land from the Vuntut Gwitchin Government (Christine Creyke, Lands Manager, Natural Resources, Vuntut Gwitchin Government, Box 94, Old Crow, Yukon Y0B 1N0). For all other locations, no specific permission was required, as these locations were not privately-owned or protected in any way at the time. The field studies did not involve endangered or protected species. Two legs of each sample were preserved in ethanol for the molecular analyses. DNA was extracted from leg tissue using the QIAGEN DNEasy Blood & Tissue Kit (QIAGEN; Hilden, Germany) following the manufacturer’s protocols.

### Mitochondrial DNA

A 1450 bp (base pairs) region of the mitochondrial gene - COI - was amplified using two sets of widely used primers LCO-HCO and Jerry-Pat. Primer sequences and PCR protocols are as in [32]. Purified products were sequenced on a Beckmann Coulter (Bromma, Sweden) CEQ 8000 capillary sequencer using forward primers, and additionally with the reverse primer when the sequence quality was not optimal. Bioedit v7.05.03 [33] was used to visualize and align the resulting chromatograms. Alignment was straightforward, with no indels. Genbank accession numbers are listed in Table S1.

A 28 bp region (bp 634–661) with ambiguous data for several sequences was excluded from further analyses; the final dataset hence consisted of 1422 bp. Haplotypes were identified and a statistical parsimony network constructed in TCS v1.21 [34], [35]. Haplotype diversity (H; the probability that two randomly chosen haplotypes in the sample are different [36] was estimated in Arlequin 3.2 [37]. Genetic differentiation in the species was estimated in Arlequin using exact tests of pairwise population differentiation [38], [39], in addition to global and among-population Ф_ST_ values. The exact tests of differentiation were each based on a Markov Chain of 100,000 steps. Populations represented by fewer than 5 individuals were not included in the above-mentioned population level analyses. We tested whether the distribution of pairwise nucleotide differences among sequences fit a model of sudden demographic expansion (mismatch distribution analysis; [40], [41]. We also performed a Fu’s Fs test of selective neutrality, where a strongly negative value of Fs indicates population demographic expansion. A total of 5000 samples were simulated in the analysis.

The phylogeny of haplotypes, including a *P. c-album* sequence (Genbank accession: JN093200) as outgroup, was inferred using maximum likelihood under the GTR+I+G model in the software RaXML v7.2.6 [42]. Bootstrap support values were calculated from 100 pseudorandom replicates.

### Microsatellites

Eight microsatellite loci developed for *P. c-album* ([19] *Polalb2*, *Polalb5*, *Polalb6*, *Polalb7*, *Polalb8*, *Polalb11*, *Polalb17*, *Polalb20;* see Protocol S1 for details about microsatellite development) were found to be suitable for use in *P. faunus*. In this study, the original PCR conditions were modified with a multiplex protocol using the Type-it Microsatellite PCR Kit (QIAGEN). All multiplex PCR reactions were performed according to manufacturer’s guidelines with an annealing temperature of 56°C and 2.0 µl DNA template. Loci *Polalb7*, *11*, *17* & *20* formed one multiplex combination, while *Polalb 2*, *5*, *6 & 8* formed a second. Primer concentrations were adjusted to normalize peak heights during electrophoresis. *Polalb10* was incompatible with other loci and hence amplified independently using the protocol described in [19]. Dye-labeled forward primers were used to estimate allele sizes by electrophoretic separation on the CEQ 8000 capillary sequencer. Alleles were scored by the peak calling software supplied with the sequencer, and manually corrected wherever necessary.

Allelic variability, global and pairwise population F_ST_ values were calculated in Arlequin. Statistical significance of pairwise F_ST_ values was tested using 100,000 permutations. Hardy-Weinberg equilibrium was tested based on exact tests with a Markov chain of 1,000,000 steps and 100,000 dememorization steps. The software STRUCTURE [43] assigns individuals to genetic populations based on multi-locus data. It relies on a model-based clustering algorithm within a Bayesian framework to infer genetic clusters (populations), and for every individual in the study estimates the proportion of ancestry from each of these populations. The user can designate the maximum number of populations (K) *a priori*. In this study, we ran consecutive analyses with increasing values of K from one to 14. Theoretically, one can examine likelihood scores from each run to determine the total number of genetic populations that best explains the data. We imposed the LOCPRIOR model with admixture [44] to improve clustering performance. Under this model i) information on the population of origin of individuals assists in the clustering exercise, and ii) individuals are allowed to draw their gene pool from more than one population. Each analysis was run for a million Markov Chain Monte Carlo steps preceded by a burnin of 100,000 steps. The dataset was reanalyzed using the same procedures after grouping the individuals into subspecies instead of populations in the LOCPRIOR model. Finally, we ran analyses with the dominant markers model ([45] by turning on the RECESSIVEALLELES option to check whether null alleles affected assignment of individuals to genetic clusters (see [19]). Results from the STRUCTURE analyses are graphically presented here using the program DISTRUCT 1.1 [46].

### *Wolbachia*

PCR-based assays using standard *wsp* (*Wolbachia* surface protein) gene primers [47] were used to check for the presence of *Wolbachia* in the *P*. *faunus* samples. If the bacterium is present in the cytoplasm of the host tissue from which DNA has been extracted, the primers amplify the *wsp* (*Wolbachia* surface protein) gene, which can be visualized electrophoretically. This technique is widely employed to detect the presence of *Wolbachia* in the host tissue, and our own assay in *P. c-album* indicates that the species has an almost 100% infection status [19]. PCR reaction protocols were as for COI, but differed in that the annealing temperature was 55°C for *wsp*. 8 µl of product was checked on a 1% agarose gel with ethidium bromide staining. Samples that did not amplify *wsp* were tested a second time. All PCR reactions included positive and negative controls (ddH2O).

## Supporting Information

Table S1 **List of samples used along with the collection localities, Genbank accession numbers, collectors and haplotypes.**
(XLS)

Protocol S1 **Protocol followed for isolation of 10 microsatellite loci for** ***Polygonia c-album*** **. Six of these loci were used in this study.**
(DOC)
