# Supplementary material for: Investigating Concordance among Genetic Data, Subspecies Circumscriptions and Hostplant Use in the Nymphalid Butterfly Polygonia faunus
Source: PLoS One. 2012 Jul 23;7(7):e41058. doi: 10.1371/journal.pone.0041058 (PMC3402543; doi:10.1371/journal.pone.0041058)
Supplement: Protocol S1 — Protocol followed for isolation of 10 microsatellite loci for Polygonia c-album . Six of these loci were used in this study. (DOC) [file pone.0041058.s002.doc]

DNA from collected butterflies was preserved by immersing two legs in alcohol. Total genomic DNA was extracted using the QIAGEN DNeasy tissue extraction kit (Qiagen, Hilden Germany). The microsatellite markers were developed by ecogenics GmbH (Zurich, Switzerland) using the following protocol: Size selected genomic DNA ligated into SAULA/SAULB-linker (Armour et al. 1994) was used to make an enriched library by magnetic bead selection with biotin labelled (CT)13, (GT)13, (GTAT)7 and (GATA)7 oligonucleotide repeats (Gautschi et al. 2000). Of 470 recombinant colonies screened, 195 showed a positive signal after hybridization (160 GT/CT, 35 GTAT/GATA). 83 plasmids from positive clones were sequenced and primers were designed for 23 microsatellite inserts, of which 19 were tested for polymorphism. Initial tests for polymorphism were performed on 10-15 individuals from the Swedish population. 5 primer pairs were excluded because they did not amplify in 3 or more individuals, whereas four primer pairs were excluded because they resulted in a complicated allelic pattern, which would be difficult to interpret.
